# Experiencing a Natural Disaster Accelerates Aging of the Immune System

**DOI:** 10.1093/geroni/igab046.2538

**Published:** 2021-12-17

**Authors:** Marina Watowich, Kenneth Chiou, Michael Montague, Melween Martínez, James Higham, Lauren Brent, Michael Platt, Noah Snyder-Mackler

**Affiliations:** 1 University of Washington, Tempe, Arizona, United States; 2 Arizona State University, Tempe, Arizona, United States; 3 University of Pennsylvania Perelman School of Medicine, Philadelphia, Pennsylvania, United States; 4 Medical Sciences Campus- University of Puerto Rico, Sabana Seca, Puerto Rico, United States; 5 NYU, New York University, New York, United States; 6 University of Exeter, Exeter, England, United Kingdom; 7 University of Pennsylvania, Philadelphia, Pennsylvania, United States

## Abstract

Extreme adverse events such as natural disasters can accelerate disease progression and promote chronic inflammation. These phenotypes also increase in prevalence with age, suggesting that experiencing adversity might accelerate aging of the immune system. Adversity can induce persistent gene regulatory changes which may mechanistically explain the immune similarities between aging and adversity. To test how immune system aging is accelerated following a natural disaster, we measured the impact of Hurricane Maria on peripheral blood immune cell gene expression in a population of free-ranging rhesus macaques (Macaca mulatta) from before (n=435) versus after (n=108) Hurricane Maria. Experiencing Hurricane Maria altered the expression of 260 genes (FDR<10%), which were primarily involved in the inflammatory response. There was significant overlap in these hurricane-affected and age-associated genes with 40% (n=104) being associated with both the hurricane and aging, more than double the expected amount (Fisher’s Exact Test OR=3.7, p=4.06 x 10–21). The effects of the hurricane and aging on gene expression were also significantly correlated (rho=0.23, p=1.33 x 10-84), suggesting that they alter similar molecular pathways in the immune system. Further, we found that animals that experienced the hurricane had a gene expression profile that was, on average, 1.6 years older than animals that did not experience the hurricane (the equivalent of 6–7 years in a human lifespan, p=0.003). Together, our results provide some of the first evidence that extreme natural disasters mechanistically accelerates aging in the immune system.

